# Structure-Based Virtual Screening of Tumor Necrosis Factor-α Inhibitors by Cheminformatics Approaches and Bio-Molecular Simulation

**DOI:** 10.3390/biom11020329

**Published:** 2021-02-22

**Authors:** Sobia Ahsan Halim, Almas Gul Sikandari, Ajmal Khan, Abdul Wadood, Muhammad Qaiser Fatmi, René Csuk, Ahmed Al-Harrasi

**Affiliations:** 1 Natural and Medical Sciences Research Center, University of Nizwa, Birkat Al-Mouz, Nizwa 616, Sultanate of Oman; ajmalkhan@unizwa.edu.om; 2 Department of Biosciences, COMSATS University Islamabad, Park Road, Chak Shahzad, Islamabad 45600, Pakistan; gul.sikandari67@gmail.com; 3 Department of Biochemistry, Abdul Wali Khan University Mardan, Khyber Pakhtunkhwa 23200, Pakistan; awadood@awkum.edu.pk; 4 Martin-Luther-University Halle-Wittenberg, Organic Chemistry, Kurt-Mothes-Str.2, D-06120 Halle (Saale), Germany; rene.csuk@chemie.uni-halle.de

**Keywords:** tumor necrosis factor–α, descriptor analysis, 2D-similarity searching, pharmacophore modelling, molecular docking, absorption, distribution, metabolism, excretion and toxicity (ADMET) prediction, molecular dynamics simulation, MM-PBSA calculation

## Abstract

Tumor necrosis factor-α (TNF-α) is a drug target in rheumatoid arthritis and several other auto-immune disorders. TNF-α binds with TNF receptors (TNFR), located on the surface of several immunological cells to exert its effect. Hence, the use of inhibitors that can hinder the complex formation of TNF-α/TNFR can be of medicinal significance. In this study, multiple chem-informatics approaches, including descriptor-based screening, 2D-similarity searching, and pharmacophore modelling were applied to screen new TNF-α inhibitors. Subsequently, multiple-docking protocols were used, and four-fold post-docking results were analyzed by consensus approach. After structure-based virtual screening, seventeen compounds were mutually ranked in top-ranked position by all the docking programs. Those identified hits target TNF-α dimer and effectively block TNF-α/TNFR interface. The predicted pharmacokinetics and physiological properties of the selected hits revealed that, out of seventeen, seven compounds (**4, 5, 10, 11, 13–15**) possessed excellent ADMET profile. These seven compounds plus three more molecules (**7, 8 and 9**) were chosen for molecular dynamics simulation studies to probe into ligand-induced structural and dynamic behavior of TNF-α, followed by ligand-TNF-α binding free energy calculation using MM-PBSA. The MM-PBSA calculations revealed that compounds **4, 5, 7** and **9** possess highest affinity for TNF-α; 8, 11, 13–15 exhibited moderate affinities, while compound **10** showed weaker binding affinity with TNF-α. This study provides valuable insights to design more potent and selective inhibitors of TNF-α, that will help to treat inflammatory disorders.

## 1. Introduction

Tumor necrosis factor-α (TNF-α), also known as cachectin or cachexin, is an important cytokine that plays a role in both pathological and physiological inflammatory processes. TNF-α is involved in several acute phase reactions as it regulates immunity. T-lymphocytes, monocytes and macrophages produces TNF-α in response to immunological reactions that provides immunity to the body. Moreover, endothelial cells, cardiac myocytes, adipose tissues, fibroblasts and neurons also produce TNF. It acts as an endogenously produced pyrogen that helps in the induction of apoptosis, fever inflammation and cachexia that inhibits viral replication and tumorigenesis [1,2,3,4,5]. The gene of TNF-α is present on chromosome 6p21.3 in human beings in close proximity to the gene of TNF-β. The gene with molecular weight 3 kb contains 4 exons. The untranslated region of TNF-α mRNA contains adenosine and uridine residues, which is also present in other cytokines and inflammatory mediator e.g., interferon, TNF-β, Granulocyte-macrophage colony-stimulating factor (GM-CSF) and interleukin-1 [1,2,4]. TNF-α mediates its inflammatory response and physiological functions by binding to its receptors, known as TNF-receptors (TNFRs). TNF-α can bind two receptors, TNFR1 (CD120a or p55/60) and TNFR2 (CD120b or p75/80) [6]. TNFR1 is expressed in most tissues and can be fully activated by both the membrane-bound and soluble trimeric forms of TNF, whereas TNFR2 is found typically in cells of the immune system and responds to the membrane-bound form of the TNF homotrimer [4]. Upon TNF-α/TNFR interaction, several pathways, including Mitogen-activated protein kinase (MAPK), NF-κB, and death signaling, are activated [7,8,9,10]. Usually, the inflammatory response occurs in joints and skin cells when TNF-α binds with TNFR and cause the release of inflammatory agents. This inflammatory mechanism is important to eliminate invaded pathogens that cause infections and pathological conditions. Moreover, several autoimmune conditions are linked with the dysregulation or elevated plasma level of TNF-α, e.g., rheumatoid arthritis, Alzheimer’s, depression, inflammatory bowel diseases, ankylosing spondylitis, psoriasis, cancer, and ulcerative colitis [11,12,13,14,15,16]. The severity of these diseases ranges from mild to chronic to fatal. The role of TNF-α is also studied in atherosclerosis and type 2 diabetes mellitus (DM) [17,18]. TNF-α plays a critical role in intermediary metabolism, hence its dysregulation may contribute in the development of type-II DM and cardiovascular diseases. Recent research studies showed that the inhibition of TNF-α helps in recovering insulin resistance and lowers lipid profile in patients with inflammatory disorders. TNF-α is also associated with the propagation of heart failure [19,20]. The study showed that patients with heart failure (HF) have increased levels of some inflammatory markers, among which TNF-α is the most prominent and linked with the progression and severity of the disease. The higher level of TNF-α give prognostic information in HF patients [21]. Apart from TNF-α, increased levels of TNF receptors (i.e., TNFR-1 and TNFR-2) have also been found in soluble forms in the circulation, that regulate the activity of TNF-α in HF patients [22]. The unresolved inflammation caused by TNF-α can result in malignancy, which can lead to carcinogenesis because of its role in the propagation of chronic inflammatory diseases. TNF-α effects are more prominent during the early stages i.e., invasion and angiogenesis versus carcinogenesis progression. Additionally, systemic administration of TNF-α showed toxic side effects e.g., organ failure and hypotension. The mechanism by which TNF-α induces and promotes tumor is based on ROS and RNS (reactive oxygen species and reactive nitrogen species) production, which damages the DNA and leads to tumorigenesis. The elevated levels of TNF-α in preneoplastic lesion have also been found in *H. pylori* positive gastric lesions. Elevated levels of TNF-α are also seen in oral squamous cell carcinoma; this eventually leads to tumorigenicity of cancer cells [23,24]. These effects of TNF-α make it a potential and emerging drug target to treat these immunological disorders [11].

The preventive therapies against TNF-α can reduce the symptoms of these pathological conditions. A significant improvement can be observed in the treatment of TNF-α related disease by impairing the interaction between TNF-α/TNF-α receptor [25], hence, targeting this protein–protein interface can be an effective therapy [26,27]. Usually, monoclonal antibodies are used against TNF-α; however, these antibodies have several side effects and have a high cost [28]. Drug discovery is a laborious task which can be foster by in silico drug designing methods [29,30,31].

The use of computational methods in drug designing has led to the discovery of several drug-like molecules which are in clinical trials [32,33]. This field has gained momentum to find novel immunomodulators against immune diseases for immunotherapeutic purposes [30,34]. This study focused on the in silico designing and development of new drug-like molecules against TNF-α to reduce the inflammation and related disorder caused by dysregulation of TNF-α.

## 2. Material and Methods

### 2.1. Descriptor Based Screening of Zinc Is Not Commercial (ZINC) Database

In the first step, forty-two known inhibitors of TNF-α (TNFI1-TNFI42) with reported IC_50_ values were selected from a literature survey (Supporting Information, Table S1) and their physicochemical properties including molecular weight, topological polar surface area (TPSA), logP (octanol/water), hydrogen bond donor atoms, hydrogen bond acceptor atoms and number of rotatable bonds were calculated (Table S2). Based on these physicochemical descriptors, ZINC database was screened, and 205,000 compounds were downloaded in SDF format from “Drug-like” subset of ZINC database [35] and imported into Molecular Operating Environment (MOE) (Chemical computing group, 910-1010 Sherbrooke St. W. Montreal, QC, Canada) [36] database, where compounds were converted into their three-dimensional (3D-) structures by Wash module. ‘WASH’ fulfils the valency of compounds by directly adding hydrogen atoms on each compound, and calculates partial charges (as per pre-defined force field; here we used Merck molecular force field-94x (MMFF-94x) and minimize the structures with the gradient of 0.1 RMS kcal/mol/Å.

### 2.2. 2D-Similarity Searching

Two-dimensional similarity searching or fingerprint-based similarity searching is the most widely used approach and efficient tool for database screening, because of its simple way of estimating the degree of structural similarity between two molecules. Those molecules which passed the descriptor-based criteria were directed to the 2D-similarity searches by using the “Tanimoto coefficient” with MACCS (Molecular Access System) structural keys (166 bits) through MOE [36]. The chemical structures of most active inhibitors were used as queries (Table S1). Subsequently, the retrieved hits were subjected to 3D-pharmacophore-based filtration.

### 2.3. Pharmacophore Modelling

Pharmacophore modelling is a powerful tool to identify the potent inhibitor of specific target. Pharmacophores are chemical features of compound that are required for protein-ligand binding for example hydrogen bonds, ionic interactions, aromatic or lipophilic contents, etc. Different pharmacophore models were generated on MOE [36] using the PPCH pharmacophore annotation scheme which has many annotation points including hydrogen bond donor/acceptor and their projections, π *vs*. non-π H-bond donor/acceptor, general π *vs*. non-π distinctions, metal ligator and its projection, cation and anion, π-ring centroid and hydrophobe point types.

### 2.4. Structure-Based Virtual Screening (SBVS)

The compounds which matched with the generated models were submitted to structure-based screening by fast rigid exhaustive docking (FRED) [37], AutoDock Vina [38], MOE [36] and Molegro virtual docker (MVD) [39]. For re-docking experiment, two protein-ligand complexes of TNF-α (PDB codes: 2AZ5 [40] and 5MU8 [41]) were used, while SBVS was performed on 2AZ5 due to its high resolution than 5MU8. Followed by SBVS, the best predicted compounds were selected through consensus strategy and their pharmacokinetic analysis was performed. Later, ten complexes were subjected to Molecular Dynamics (MD) simulations, followed by a ligand-TNF-α binding energy calculation with a molecular mechanics Poisson–Boltzmann surface area (MM-PBSA) approach.

#### 2.4.1. FRED

For FRED (OpenEye Scientific, 9 Bisbee Court, Santa Fe, NM 87508, USA) docking, the compounds database was created by OMEGA [42] which generates the conformations of each compound. The protein files were prepared by FRED receptor software. The active site was defined for docking by the bound ligand and the box volume was set to 2173Å, while inner and outer contours were adjusted to 78 Å and 1120 Å, respectively. Docking was performed by FRED. ChemGauss4 scoring function was used, which is a Gaussian scoring function that provides favorable values under those conditions in which there is little volume overlap and proteins and ligands have high surface contact. For each ligand, thirty docked poses were retained as default parameters.

#### 2.4.2. AutoDock Vina

The compounds were docked by AutoDock Vina (version 1.1.2, Scripps Research, San Deigo, California Jupitar, Florida, USA) using PyRx—Virtual Screening Tool (https://pyrx.sourceforge.io/ (accessed on 18 February 2021)). AutoDock Vina requires the receptor and the ligand files in PDBQT (protein data bank, partial charge (Q) and atom type (T)) file format. The PDB files of receptors and the mol2 files of ligands were converted to PQBQT format by PyRx. For VS, docking search space was defined around 3 Å of co-crystallized ligand with a grid box size of 70 Å × 70 Å × 70 Å points and grid spacing of 0.375 Å. The numbers of binding modes and exhaustiveness of search were set to 30 and 8, respectively. The Lamarckian genetic algorithm and empirical energy function were used for docking. By default, ten docked conformations were generated for each docked ligand.

#### 2.4.3. Molecular Operating Environment (MOE)

MOE docking suit was used for virtual screening (VS) using the virtual screening protocol of MOE in combination with the triangle matcher docking algorithm and London dG scoring function. The active site was defined by selecting the co-crystalized ligand in the protein. By default, thirty docked conformations of each ligand were retained after docking.

#### 2.4.4. Molegro Virtual Docker

The protein and ligand structures were imported into the MVD (version 2019.7.0.0, Molexus IVS, Rørth Ellevej 3, Rørth, DK-8300 Odder, Denmark) workspace in “pdb” and “mol2” format, respectively. The ligand files were prepared as per MVD protocol, which applies charges and explicit hydrogen, and the valences, bond orders, and protons were corrected in the ligands. The ligand binding site in the protein was defined by cavity detection algorithm of MVD. Thirty docking runs were performed for each ligand with a maximum iteration of 1500 and maximum population size of 50, which showed thirty docked poses of each ligand after docking.

### 2.5. Selection of Best Binders by Consensus Approach

After VS, the docked libraries from each program were sorted on the basis of docking scores, and the top ranked compounds from each library were selected. Those compounds which were ranked mutually in top 100 position by all the docking programs, were declared as potential ‘Hits’ or ‘Binders’. The most probable binding orientation of the selected hits were analyzed through conformational sampling of docked poses given by each docking program.

### 2.6. ADMET Prediction

The pharmacokinetic (absorption, distribution, metabolism, excretion and toxicity (ADMET)) properties of selected hits were predicted by SwissADME server (http://www.swissadme.ch/ (accessed on 18 February 2021)) [43] and ADMETsar (http://lmmd.ecust.edu.cn/admetsar1/predict/ (accessed on 18 February 2021)) [44]. SwissADME calculates physicochemical descriptors, predicts ADME parameters, pharmacokinetics properties, and the drug-likeness of small molecules, while ADMETsar can predict ~fifty ADMET properties with quantitative structure activity relationship (QSAR) models.

### 2.7. MD Simulations Protocols

Molecular dynamics (MD) simulations were carried out by the NAMD program (Theoretical and Computational Biophysics Group, NIH Center for Macromolecular Modeling and Bioinformatics, Beckman Institute, University of Illinois at Urbana-Champaign, Illinois, USA) [45]. Binding energies of compounds were calculated by Amber package (Dept. of Pharmaceutical Chemistry, University of California, San Francisco, CA, USA) [46]. For MD simulations, the ff14SB amber force filed and general amber force field (GAFF) were used for TNF-α and ligands, respectively. Antechamber was used to generate files for ligands. The xleap program [46] was used to create topology/parameter (.prmtop) file and a coordinate (.inpcrd) file of TNF-α. All systems were electronically neutralized by the addition of 4 Na^+^ ions. TIP3P water box of 12 Å size, as measured from the edge of TNF-α, was used to explicitly solvate both protein and its complexes. A standard protocol was followed to energy minimize and equilibrate the systems prior to MD simulations using NAMD. The water equilibration was continued until both temperature and total energy were converged. The periodic boundary conditions (PBCs) were used throughout 25 ns long MD simulations for each system. The SHAKE algorithm [47] was used to control/fix all bonds involving hydrogen; the time step was set to 2 fs. The cutoff for van der Waals and electrostatics was set to 14 Å, with a switching function of 2 Å. The particle mesh Ewald (PME) method was applied for long range electrostatics [48]. The isothermal-isobaric (NPT) ensemble was used to keep the system at 310 K temperature and 1 atm pressure using Langevin thermostat and Nose–Hoover Langevin piston method, respectively. The resulting trajectories were written at every 1 ps. Molecular visualization was performed through the visual molecular dynamics (VMD) program Theoretical and Computational Biophysics Group, NIH Center for Macromolecular Modeling and Bioinformatics, Beckman Institute, University of Illinois at Urbana-Champaign, Illinois, USA) [49]. R program was used to calculate root mean square deviation (RMSD), root mean square fluctuation (RMSF), and radius of gyration (Rg).

### 2.8. Binding Free Energy Calculation

The binding energy of ligand-protein association was calculated with MM-PBSA (Dept. of Pharmaceutical Chemistry, University of California, San Francisco, CA, USA) [50], where the binding free energy was decomposed into the relative free energy of the solvated receptor/protein–ligand complex and the separated, solvated ligand and receptor, as given in Equation (1):

(1) $$∆G_{binding}=\;\langle G_{protein-ligand\;complex}\rangle \;-\;\langle G_{Protein}\rangle \;-\langle G_{ligand}\;\rangle$$

**Figure 1:**
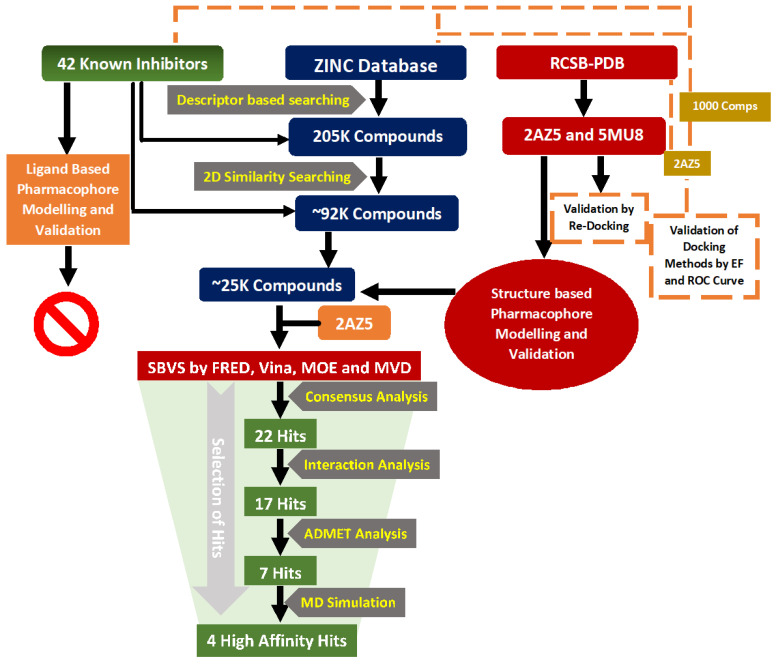
The schematic presentation of computational workflow.

Each free energy change in Equation (1) can be calculated by summing up several other terms, such as (i) the molecular mechanics energy including electrostatics and van der Waal’s contributions, (ii) the polar solvation free energy estimated from the Poisson-Boltzmann (PB) model, (iii) the non-polar solvation energy obtained from a linear relation to the solvent accessible surface area (SASA), and (iv) a cavity dispersion term. The complete computational workflow is depicted in Figure 1.

## 3. Results and Discussion

### 3.1. Ligand Based Virtual Screening

**Figure 2:**
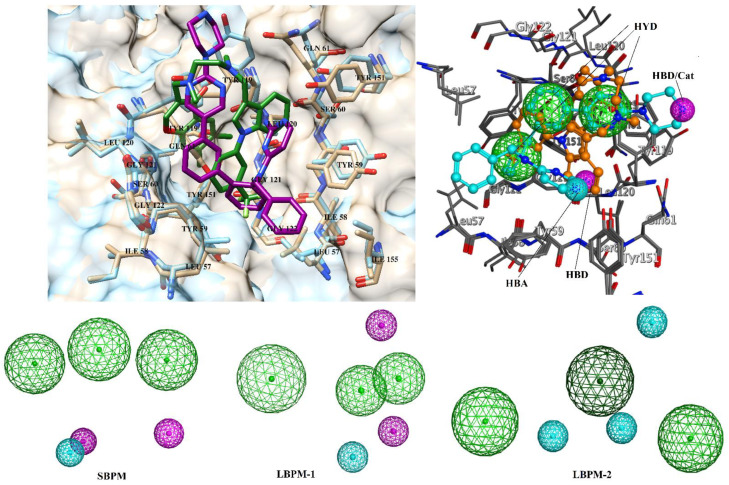
Superimposed view of 2AZ5 and 5MU8. The ligands are shown in green and magenta sticks. The active site residues are labelled. The structure-based pharmacophore model (SBPM) and ligand-based pharmacophore models (LBPM1 and LBPM2) are shown. Hydrophobic feature (HYD), hydrogen bond donor (HBD) and hydrogen bond acceptors (HBA) are shown in green, magenta and cyan spheres, respectively.

The “drug-like” subset of ZINC database was filtered according to the physicochemical descriptors calculated from forty-one known inhibitors of TNF-α. Compound TNFI32 (Table S1) was not used in descriptor calculation due to its high molecular weight. The descriptors were molecular weight = 269–549, total polar surface area = 32.59–176.51, number of hydrogen bond acceptor atoms = 2–10, number of hydrogen bond donor atoms = 0–5, number of rotatable bonds = 1–12, and logP (log octanol/water partition coefficient) = −1–5.96. Subsequently, 205,000 molecules passed the descriptor-based criteria, which were further filtered through 2D similarity searching method. Moreover, 2D similarity searching was carried out on MOE with Molecular Access System (MACCS) structural keys with the general form of Tanimoto coefficient [51]. MACCS consists of 166 structural substructures or patterns with 1–10 non-hydrogen atoms. As a default parameter, 85% of overlap was selected. The chemical structures of the five most active inhibitors of diverse structures with IC_50_ values ranging from 0.004 µM to 0.06 µM, were used as queries (Table S1), where compounds TNFI5, TNFI9, TNFI24, TNFI39, and TNFI41 were returned with the best Tanimoto values ranging from 0.66 to 0.88. Subsequently, 92,247 compounds passed 2D similarity searching. Followed by two-fold filtration, pharmacophore modelling was performed to further screen ~92 K compounds. One structure-based pharmacophore model (SBPM) and two ligand-based models (LBPM1 and LBPM2) were generated. SBPM was generated by aligning two X-ray crystallographic structures of TNF-α in complex with cognate inhibitors (2AZ5, resolution = 2.10 Å and 5MU8, resolution = 3.0 Å) by MOE. SBPM comprises six pharmacophoric features including three hydrophobic (HYD), one hydrogen bond donor (HBD), one hydrogen bond acceptor (HBA) and one cation (CAT). LBPM1 was constructed by aligning six most active TNF-α inhibitors (TNFI2, TNFI5, TNFI6, TNFI22, TNFI24 and TNFI39), using MOE (Figure S4). The compounds were aligned by flexible alignment module of MOE which align structurally diverse molecules through stochastic conformational search, while LBPM2 was generated by aligning twenty active TNFIs (TNFI1–11, TNFI15, TNFI20–25, TNFI34 and TNFI41) with pIC_50_ values in a range of 6–8 (Figure S4). LBPM1 was composed of three HYD, one HBA and two HBD, while LBPM2 possess three HYD and three HBA features. The ability of each model to extract active molecules from decoy compounds was examined by embedding forty-two known inhibitors in randomly chosen 1000 compounds from the ZINC database. SBPM, LBPM1 and LBPM2 accurately retrieved 37/42, 22/42, and 23/42 known inhibitors, respectively. Thus, SBPM, LBPM1 and LBPM2 showed 88.10%, 52.38% and 54.76% enrichment results. Based on superior screening ability, SBPM was selected for the filtration of ~92 K compounds. Moreover, ~25,000 compounds were matched with at least four features of SBPM, that were targeted to TNF-α/TNFR binding interface by structure-based molecular docking protocol. All the pharmacophore models are shown in Figure 2.

### 3.2. Structure-Based Virtual Screening

**Figure 3:**
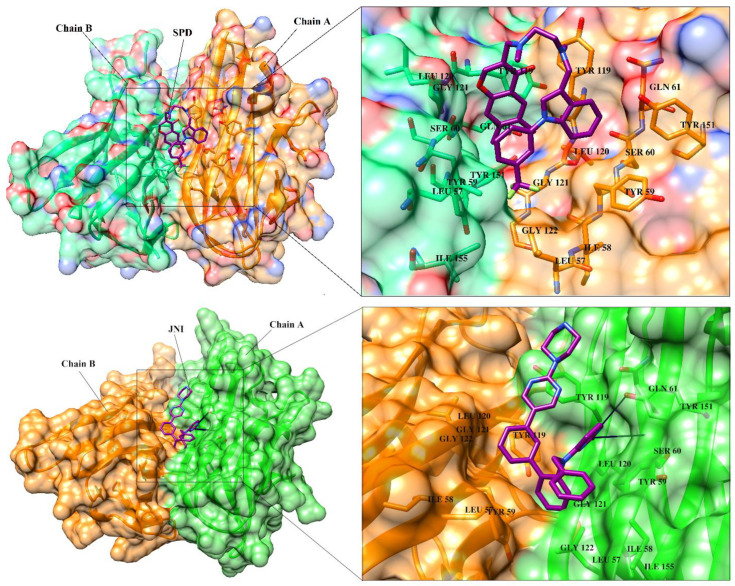
The 3D-structure of TNF-α is presented in dimeric form in complex with SPD (A) and JNJ525 (B). The ligand binding residues at dimer interface are highlighted. Chain A and B are presented in orange and green color, respectively. The binding site residues are depicted in stick model in their respective chain colors. The co-crystallized ligands (SPD and JNJ525) are shown in magenta color (stick model). SPD is predominantly bound with hydrophobic interactions, however JNJ525 is bound with hydrophobic interactions as well as H-bonding with Ser60 and Tyr151. Hydrogen bonds are depicted in black lines.

SBVS was carried out via a multi-docking approach using four different docking methods (FRED, AutoDock Vina, MOE and MVD) in order to select the most appropriate compounds for TNF-α with high binding affinity. Prior to vs. of ~25K molecules, each docking method was validated by the re-docking of co-crystallized ligands present in the X-ray crystal structure of human TNF-α (PDB code: 2AZ5 and 5MU8). TNF-α is a trimeric protein, however, only two of its subunits contribute in the formation of receptor binding site. The receptor binding surface is composed of sixteen contact residues including 6 tyrosine residues. Out of 16, nine residues are present on chain A and seven are located on the chain B. These residues are potential target for ligand binding. The receptor binding residues are Leu57(A/B), Tyr59(A/B), Ser60(A/B), Gln61(A), Tyr119(A/B), Leu120(A/B), Gly121(A/B), Gly122(A), Tyr151(A/B). These residues provide both hydrophilic and hydrophobic interactions to ligand to stabilize its binding at TNFR binding site. The receptor binding site in complex with co-crystallized ligand is depicted in Figure 3. The re-docked orientations of co-crystallized ligand 1 (SPD) and co-crystallized ligand 2 (JNJ525) were found to be similar to their X-ray conformations. The docked conformation of SPD was returned with RMSD values of 1.60 Å, 2.65 Å, 1.28 Å and 2.76 Å by FRED, AutoDock Vina, MOE and MVD, respectively, while the re-docked pose of JNJ525 showed RMSD values of 1.57 Å, 1.54 Å, 1.99 Å and 1.31 Å for MOE, FRED, AutoDock Vina, and MVD, respectively (Figure S1). The results are acceptable, since the RMSD ≤ 3.0 Å is usually considered satisfactory in re-docking experiments, therefore, the applied docking parameters can be used in the virtual screening (VS) experiments. Moreover, the VS accuracy of all the programs was scrutinized by correct identification of known inhibitors out of decoys set. For this purpose, the selected TNFIs (Table S1) were embedded in a dataset of 10,000 decoys (chosen from ZINC) and docked by all the programs. The results were examined by enrichment factor (EF) and Receiver operating characteristic-curves (ROC-curves). EF is a widely used metric for the comparison of VS results. EF was calculated by Equation. 2, while %EF for program was calculated by Equation (3). Success was declared when 50% of TNFIs were ranked by docking programs in the top 1% (top 100 compounds), 5% (top 500 compounds) and 10% (top 1000 compounds) of screened library.

(2) $$\begin{array}{cc} Enrichment\;Factor= & \frac{HITS\;sampled/HITS\;total}{N\;sampled/N\;total} \end{array}$$

*N total* = total number of compounds in the database, *N sampled* = number of ligands in the docked database to be examined, *HITS total* = total number of active compounds, *HIT sampled* = number of active inhibitors found in top *N sampled* ligands of docked library.

(3) $$\begin{array}{cc} \%\;Enrichment\;Factor= & \frac{Enrichment\;Factor}{Ideal\;Enrichment\;Factor} \end{array}\;\times \;100$$

The optimal threshold for VS accuracy was justified by the following criteria: if in top 1%, in top 5% and in top 10% of screened database, all the 42 TNFIs are successfully identified, then the ideal EF would be 100, 20, and 10, respectively. All the docking methods effectively discriminated TNFIs in the decoys set. FRED, MOE, Autodock Vina and MVD showed >21, >11, >28, >14%EF, respectively, in top 1% of the screened library. However, in top 5% of the docked database, >57, >23, >59 and >28%EF were obtained for FRED, MOE, Autodock Vina and MVD, respectively. The VS performance of each program was drastically improved and FRED, MOE, Autodock Vina and MVD identified >88%, >64%, > 71% and 50% of active TNFIs in top 10% screened library. The results show that each program effectively identified > 50% of TNFIs in the top 10%. The calculated %EF for each program is shown in Figure S2.

ROC curves [52] effectively differentiate between two populations (active inhibitors and decoy compounds), therefore it is used to evaluate VS performance. ROC curve describes the trade-off between sensitivity (detection of true positives by model) and specificity (ability of model to avoid false negatives). Enrichment is quantified by calculating area under the curve (AUC). If the value of AUC is ≥ 0.9, the scoring function would be considered as excellent, while the value of AUC ≤ 0.6 depicts least or no enrichment. The AUC value of 0.95, 0.86, 0.92 and 0.87 was observed for FRED, MOE, Autodock Vina, and MVD, respectively. The results depict that all the docking methods effectively discriminate TNFIs with the decoy compounds. The ROC curve of each scoring function is depicted in Figure S3. The results are encouraging; therefore, all the docking programs were employed in SBVS of ~25 K compounds.

### 3.3. Selection of Best Inhibitors

SBVS of ~25 K molecules was carried out by all the used docking programs. The most potential inhibitors of TNF-α were selected by consensus results of all the docking approaches. The docked library of each program was ranked on the basis of docking scores. Those compounds which were ranked in top 100 of docked library, were selected for further analysis. This comparison led us to choose twenty-two compounds that were ranked on the top-ranking position by all four docking methods. The molecular interactions of the selected twenty-two compounds were analyzed by Chimera [53]. The hydrogen bonding and hydrophobic interactions were visualized, which suggested that out of twenty-two, seventeen compounds are the most potential hits. The molecular structures of selected hits and their docking results are presented in Table 1.

### 3.4. ADMET Prediction

The absorption, distribution, metabolism, excretion, and toxicity (ADMET) properties of the selected hits (seventeen compounds) were predicted by SwissADME and ADMETsar. The results are tabulated in Table S3, supporting information. The results depict that all the compounds fulfil the criteria of Lipinski drug likeness and their synthetic feasibility is good. The human intestinal absorption of all compounds is high, suggesting that they can act as orally bioavailable drugs. All compounds can cross the blood–brain barrier except compounds **8, 9, 13**, and **14**. Only compounds **10** and **15** show positive results in AMES toxicity test, while the rest of the compounds are non-AMES toxic. The predicted acute oral toxicity also shows that these compounds belong to class III category, which includes compounds with LD_50_ values >500 mg/kg but <5000 mg/kg. This means that these compounds do not possess acute oral toxicity up to the concentration of 500 mg/kg, and thus, are regarded as safe. Moreover, none of the compounds is carcinogenic, indicating that these compounds possess good drug-likeness. The calculated rat acute toxicity also shows that these compounds are not lethal up to the concentration of >2 mol/kg, hence categorized as non-toxic. SwissADME also predicts skin permeability of the molecules. The predicted values of these compounds are highly negative, which means that these compounds are not permeable through skin. The more negative the log Kp (with Kp in cm/s), the less skin permeant is the molecule. Hence, we can say that these compounds can have permeability through intestine. The calculated physicochemical properties also show that the compounds possess good to moderate solubility in water, while they show moderate partition coefficient between *n*-octanol and water (log Po/w). The pharmacokinetic profile also predicts the ability of a molecule to act as a substrate/non-substrate/inhibitor/non-inhibitor of cytochromes P450 (CYP) enzyme system. The selectivity of the selected compounds is shown in Table S3. The CYP superfamily is involved in drug elimination through metabolic biotransformation. The knowledge about compounds of being substrate or non-substrate of glycoprotein (P-gp, the most important member among ATP-binding cassette transporters or ABC-transporters) is the key to appraise active efflux through biological membranes (from the gastrointestinal wall to the lumen or from the brain) [54]. P-gp also protects the central nervous system (CNS) from xenobiotics [55]. Moreover, P-gp is over-expressed in some tumor cells and leads to multidrug-resistant cancers [56]. The predicted ADMET profile shows that, out of seventeen hits, compounds **4, 5, 10, 11, 13–15** are not substrate of P-gp, therefore, these compounds (**4, 5, 10, 11, 13–15**) are regarded as good inhibitors of TNF-α/TNFR interaction.

### 3.5. Ligand-Induced Structure and Dynamics

**Figure 4:**
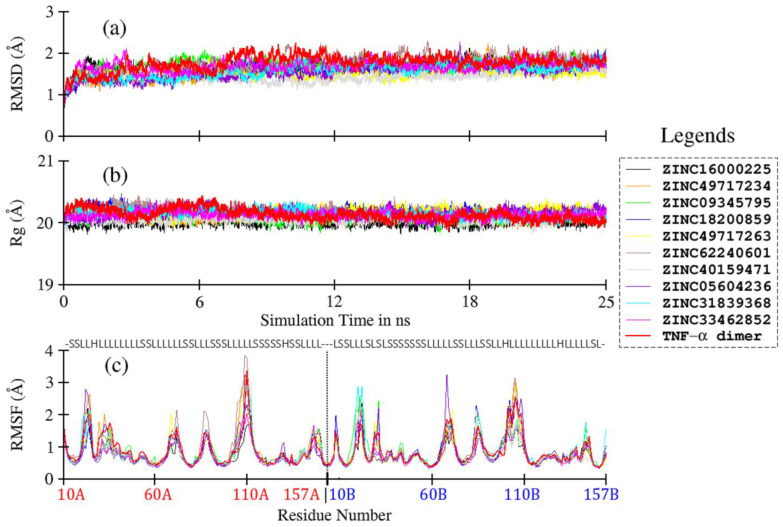
Comparative plots of root mean square deviation (**a**), radius of gyration (**b**), and root mean square fluctuation (**c**) based on Cα atoms of TNF-a dimer and its various complexes with ZINC ligands.

In order to understand ligand-induced changes in TNF-α, and vice versa, MD simulation studies were carried out for TNF-α dimer and its complexes with compounds **4, 5, 10, 11, 13–15**. Compounds **7, 8** and **9** were further considered for simulation studies due to their diverse scaffold. The resultant trajectories were analyzed in terms of RMSD, RMSF, and Rg, and plotted in Figure 4. The Cα-based RMSD of TNF-α and its complexes are plotted in Figure 4a; the average values are given in Table 2. The RMSD plots show very stable curves for all systems during the simulation time, indicating the stability of the system. For ligand-free TNF-α dimer, the average RMSD is calculated as 1.77 ± 0.19 Å. Upon ligand binding, the average RMSD values of TNF-α dimer are slightly reduced, with comparatively lower standard deviation values, ranging from 1.45 to 1.75 Å. This observation indicates minor ligand-induced conformational restrictions in TNF-α. The effect is comparatively more pronounced in **compound 11**, moderately in compounds **5, 8, 9, 13**, and **14**, and the least in compounds **4, 7, 10** and **15**, as compared to the ligand-free protein (Table 2).

The compactness of the protein system during MD simulation is reflected by Rg. The Rg values for ligand-free TNF-α dimer and its complexes are plotted in Figure 4b. Overall, no major change is observed in Rg values, as inferred from the mean values, which range from 20.01 to 20.18 Å for both ligand-free and ligand-bound TNF-α dimers (Table 3). The Cα-based RMSF plots of TNF-α and its complexes are shown in Figure 4c. Overall, all systems follow a similar RMSF trend as that of ligand-free TNF-α dimer. Most of the loop regions show higher fluctuation due to higher conformational degree of freedom, while the β-sheets remain highly rigid throughout the simulation time due to strong H-bonding, as expected. No major impact of ligand on TNF-α is observed, except for slight loop fluctuations in compound **10**, which shows slightly higher value for chain A residues (Figure 4c, brown line), and for compounds **7** (green line), **8** (blue line) and **14** (cyan line), which show higher fluctuation in chain B. Compounds **5** (orange line), **9** (yellow line) and **13** (purple line), however, exhibit slightly higher loop fluctuation in both chains A and B. Briefly, both the structural and dynamic behaviors of ligands–protein complexes show negligible ligand-induced conformational changes in TNF-α.

### 3.6. MM-PBSA Binding Energy Calculations

The strength of ligand-TNF-α association was calculated using MMPBSA methods. MM-PBSA results (Table 3) ranked the compounds in the following order: **7** < **9** < **4** < **5** < **8** < **13** < **14** < **15** < **11** < **10**, where the non-polar solvation free energies contributed favorably in all cases, as did electrostatic and van der Waals forces. The polar solvation free energy contributions to ligand binding (EPB) remained significantly unfavorable in all complexes, indicating that the major driving forces for ligand binding were from van der Waals (VDW) interactions, with few contributions from electrostatic interactions and non-polar solvation free energies (ENPOLAR), except for compound 10, where electrostatic was dominant over VDW, however, it was largely compensated by unfavorable polar solvation energy. Compounds **4**, **5**, **7** and **9** showed the highest affinity for TNF-α, exhibiting mean binding energy values of −31.43 ± 3.49, −30.37 ± 3.97, −35.23 ± 3.15 and −32.16 ± 4.06 kcal/mol, respectively. Although, the van der Waal’s energy is a major contributory factor, electrostatics also play a role in compounds **4** and **9**. Compounds **8**, **11**, **13**, **14**, **15** exhibited moderate affinities with TNF-α with MMPBSA binding energy ranging from −27.06 to −20.83 kcal/mol. Compound **10** exhibited least binding free energy value (−14.75 ± 2.99) among all ten **compounds**, indicating comparatively weaker binding affinity with TNF-α. The major energy contribution arose from the electrostatics term due to its salt nature, and therefore, the penalty for polar solvation free energy is very high.

### 3.7. Interactions Analysis of Compounds **4, 5, 7** and **9**

**Figure 5:**
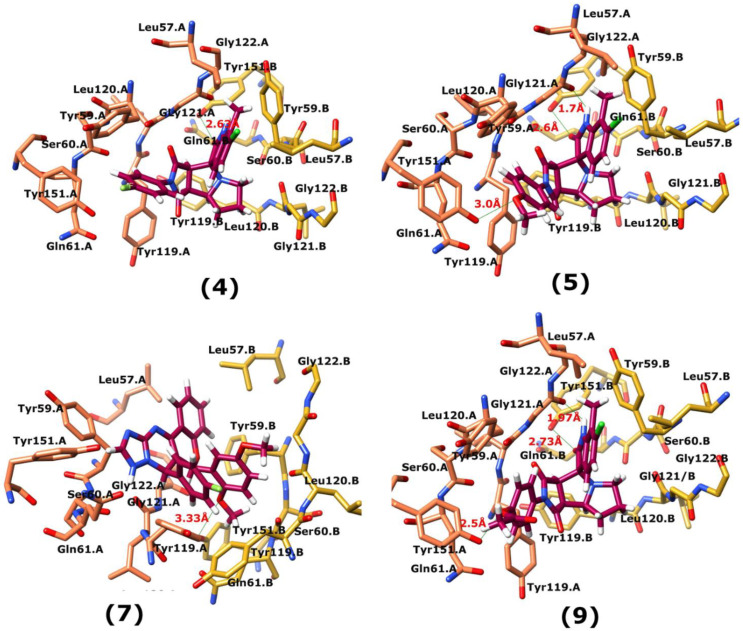
The binding interactions of compounds **4, 5, 7** and **9**. The interacting residues of chain A and B are depicted in Coral and Yellow color, respectively. The compounds are shown in magenta color (stick model). Hydrogen bonds are presented in green lines. The H-bond distances are labelled in red color. Compound’s numbers are written in parenthesis.

The binding mode analysis of compound **4** revealed that the chloro-methyl substituted indolinone ring of 4 formed H-bond with the –OH of Tyr151B (2.63 Å). However, hexahydropyrrolo-pyrrolizine-dione moiety interacts with Tyr119A, Tyr119B, Leu120B and Gly121B via HYD interactions, while the fluorobenzene ring is oriented towards chain A and surrounded by Tyr59A, Ser60A and Tyr151A. **Compound 5** interacts with the side chains of Tyr151A, Gly122A and Tyr151B via multiple H-bonds. The H-bond distance between the –OH of Tyr151A and the phenyl-substituted methoxy group is 3.0 Å. The pyrrolone moiety of compound mediates bi-dentate interactions with Gly122A (1.7 Å) and Tyr151B (2.6 Å). Moreover, several residues including Tyr119A, Tyr59B, and Tyr119B provide HYD interactions. The pyrimide moiety of compound **7** is H-bonded to the peptide carbonyl of Leu120B (2.24 Å) and interacts with the chain of Tyr119A via HYD interaction. The dimethoxy-phenyl moiety of 7 is stabilized by Leu57B and Tyr59B. Compound **9** forms three H-bonds with the B chain residues. The triazole-substituted methanium mediates bidentate interaction with carbonyl group of Ser60B (2.16 Å) and Leu120B (2.47 Å). Moreover, –OH of Tyr151B donates H-bond to the triazole nitrogen. Furthermore, the methoxy phenyl ring, and triazole substituted phenethyl ring of the compound mediates π-π interaction with Tyr119A, and CH3-π interaction with Leu57A and Leu57B, respectively. Hence, these strong H-bonds and HYD interactions provide a path to interact with the protein. The binding modes of compounds **4, 7** and **9** are depicted in Figure 5. The H-bond distances are written in the figure. The binding mode analysis suggests that the selected hits binds at TNF-α/TNFR binding interface with strong H-bonds and multiple HYD interactions, hence they are capable of hindering the interface, and can block the TNF-α activity. The docked view and binding interactions of compounds **1–3, 6, 8, 10–17** are described in the supporting information (Figure S5).

## 4. Conclusions

The current study was conducted with the aim to develop and validate the computational protocols to be employed in the designing of inhibitors against the protein–protein interfaces (PPIs), in particular, TNF-α. These PPIs have a complex nature and are challenging drug targets, which makes them difficult to handle with the computational strategies as well as experimental drug design processes. Hence, the current study comprising ligand and structure-based drug designing approach to find inhibitors against TNF-α. The combined ligand and structure-based strategy provided twenty-two potential compounds from ZINC database; among them, seventeen hits were found with higher binding potential with TNF-α. Therefore, these compounds can inhibit the over activity of TNF-α by blocking the binding of TNF-α with TNFR. However, by ADMET profiling, seven compounds were identified as excellent druglike molecules. MD simulation studies further screened the compounds, which indicated that compounds **4, 5, 7** and **9** can act as potential inhibitors of TNF- α.

## Figures and Tables

**Table 1:** The chemical structures and docking results of 17 Hits.

Compounds	ZINC ID	Chemical Structure	FRED Score	FRED Rank	Vina Score	Vina Rank	MOE Score	MOE Rank	MVD Score	MVD Rank
**1.**	ZINC04914424	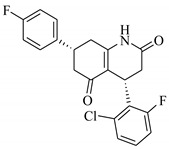	−89.32	1	−13.74	2	−23.65	5	−90.05	8
**2.**	ZINC17090251	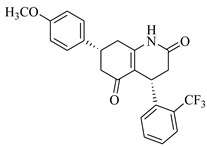	−85.29	2	−13.93	1	−20.23	11	−81.23	24
**3.**	ZINC04914300	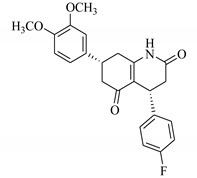	−84.08	3	−12.77	3	−19.01	24	−74.56	41
**4.**	ZINC16000225	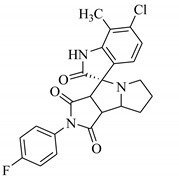	−82.55	5	−12.34	7	−23.44	8	−89.88	11
**5.**	ZINC49717234	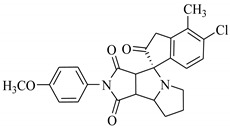	−80.65	10	−12.56	5	−26.88	1	−90.89	4
**6.**	ZINC00785143	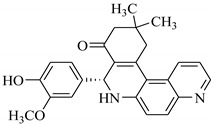	−80.26	11	−12.58	4	-20.05	15	−77.73	35
**7.**	ZINC09345795	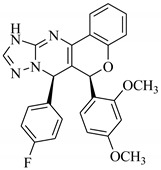	−79.69	13	−11.52	48	−18.98	29	−89.09	12
**8.**	ZINC18200859	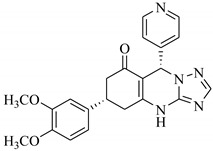	−79.68	14	−11.81	26	−20.11	12	−80.6	29
**9.**	ZINC49717263	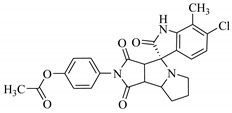	−79.25	16	−12.48	12	−22.65	9	−85.64	17
**10.**	ZINC62240601	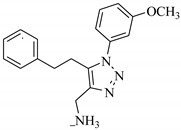	−77.64	23	−12.10	10	−16.01	55	−72.65	46
**11.**	ZINC40159471	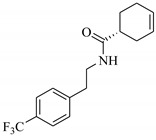	−76.00	29	−11.66	35	−24.59	4	−91.05	2
**12.**	ZINC31819567	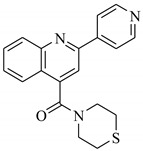	−75.81	30	−12.33	8	−15.05	89	−90.85	5
**13.**	ZINC05604236	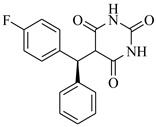	−75.50	34	−11.68	33	−15.9	63	−81.24	25
**14.**	ZINC31839368	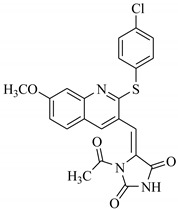	−75.19	36	−11.88	20	−15.55	71	−80.56	30
**15.**	ZINC33462852	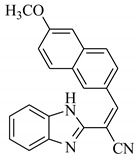	−75.05	37	−12.48	6	−16.23	45	−90.01	9
**16.**	ZINC04214135	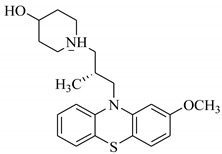	−74.89	38	−11.58	41	−19.89	18	−74.22	42
**17.**	ZINC04914400	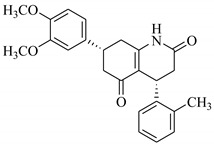	−74.84	39	−11.85	22	−19.23	21	−85.22	19

**Table 2:** Cα-RMSD and Cα-Rg of protein and its complexes, obtained by MD trajectory analysis.

Compounds	ZINC ID	RMSD (Å)	Rg (Å)
**4**	ZINC16000225	1.70 ± 0.13	20.01 ± 0.06
**5**	ZINC49717234	1.59 ± 0.17	20.10 ± 0.06
**7**	ZINC09345795	1.73 ± 0.15	20.08 ± 0.06
**8**	ZINC18200859	1.63 ± 0.15	20.18 ± 0.07
**9**	ZINC49717263	1.53 ± 0.12	20.18 ± 0.07
**10**	ZINC62240601	1.75 ± 0.20	20.17 ± 0.08
**11**	ZINC40159471	1.45 ± 0.14	20.05 ± 0.06
**13**	ZINC05604236	1.55 ± 0.18	20.16 ± 0.07
**14**	ZINC31839368	1.58 ± 0.17	20.15 ± 0.07
**15**	ZINC33462852	1.70 ± 0.14	20.13 ± 0.06
	TNF-α dimer	1.77 ± 0.19	20.14 ± 0.09

**Table 3:** Estimated protein–ligand binding energy (in kcal/mol) with MM-PBSA, as calculated from 25 ns MD trajectory. The individual contribution forming Van der Waal’s (VDW), electrostatics (EEL), polar solvation free energy (EPB) and non-polar solvation free energy (ENPOLAR) is also given.

Compounds	ZINC ID	Binding Energy ± Standard Deviation (kcal/mol)	VDW	Electrostatics	EPB	ENPOLAR
**4**	ZINC16000225	−31.43 ± 3.49	−43.07	−15.72	30.96	−3.60
**5**	ZINC49717234	−30.37 ± 3.97	−46.47	−9.91	29.89	−3.88
**7**	ZINC09345795	−35.23 ± 3.15	−48.51	−6.43	23.64	−3.93
**8**	ZINC18200859	−27.06 ± 3.93	−41.82	−6.91	25.22	−3.55
**9**	ZINC49717263	−32.16 ± 4.06	−47.36	−12.14	31.30	−3.97
**10**	ZINC62240601	−14.75 ± 2.99	−24.44	−66.08	78.55	−2.78
**11**	ZINC40159471	−20.83 ± 3.08	−30.42	−2.54	15.13	−3.00
**13**	ZINC05604236	−26.87 ± 2.67	−38.87	−14.68	29.78	−3.10
**14**	ZINC31839368	−24.40 ± 3.74	−39.63	−6.03	24.99	−3.73
**15**	ZINC33462852	−22.66 ± 2.93	−36.62	−8.87	26.28	−3.45

## Data Availability

Data is contained within the article or supplementary material.

## Supplementary Materials

The following are available online at https://www.mdpi.com/2218-273X/11/2/329/s1, The chemical structures and biological activities of selected known inhibitors of TNF-α (TNFI) (Table S1), the physicochemical properties of selected TNFIs (Table S2), the re-docked orientation of SPD and JNJ525 obtained by MOE, FRED, AutoDock Vina, and MVD (Figure S1), Percent Enrichment Factor (%EF) calculated for four docking programs on 2AZ5 (Figure S2) and Receiver Operating Curve (ROC) for 2AZ5 obtained from FRED, MOE AutoDock Vina, and MVD (Figure S3), and Pharmacokinetic and Physiological Properties of 17 selected hits (Table S3), aligned view of **compounds** TNFI2, TNFI5, TNFI6, TNFI22, TNFI24 and TNFI39 on LBPM1 and twenty Compounds (TNFI1–11, TNFI15, TNFI20–25, TNFI34 and TNFI41) on LBPM2 (Figure S4) and binding mode analysis of compounds **1-3, 6, 8, 10-17** (Figure S5) are available in supporting information.

## Abbreviations

ADMET	Absorption, Distribution, Metabolism, Excretion and Toxicity
FRED	Fast Rigid Exhaustive Docking
MD Simulation	Molecular Dynamics Simulation
MM-PBSA	Molecular Mechanics: Poisson Boltzmann Surface Area
MOE	Molecular Operating Environment
MVD	Molegro Virtual Docker
NAMD	Nanoscale Molecular Dynamics
TNF-α	Tumor Necrosis Factor-α
TNFR	Tumor Necrosis Factor-α Receptor
VS	Virtual Screening
ZINC	Zinc Is Not Commercial
