# Supplementary material for: Structure-Based Virtual Screening of Tumor Necrosis Factor-α Inhibitors by Cheminformatics Approaches and Bio-Molecular Simulation
Source: Biomolecules. 2021 Feb 22;11(2):329. doi: 10.3390/biom11020329 (PMC7926523; doi:10.3390/biom11020329)
Supplement: Supplementary file 1 [file biomolecules-11-00329-s001.pdf]

Supporting Information

Structure based Virtual Screening of Tumor Necrosis Factor-α Inhibitors by Cheminformatics Approaches and Bio-Molecular Simulation

Sobia Ahsan Halim[1]*, Almas Gul Sikandari[2], Ajmal Khan[1], Abdul Wadood[3], Muhammad Qaiser Fatmi[2]*, René Csuk[4], Ahmed Al-Harrasi[1,*]

[1]Natural and Medical Sciences Research Center, University of Nizwa, Birkat Al-Mouz, Nizwa-616, Sultanate of Oman, [2]Department of Biosciences, COMSATS University Islamabad, Park Road, Chak Shahzad, Islamabad-45600, Pakistan, [3]Department of Biochemistry, Abdul Wali Khan University Mardan, Mardan, Pakistan, [4]Martin-Luther-University Halle-Wittenberg, Organic Chemistry, Kurt-Mothes-Str.2, D-06120 Halle (Saale), Germany.

***Corresponding Authors:**

Sobia Ahsan Halim: sobia_halim@unizwa.edu.om
Ahmed Al-Harrasi: aharrasi@unizwa.edu.om
M. Qaiser Fatmi: qaiser.fatmi@comsats.edu.pk

Contents

Table S1. The chemical structures and biological activities of selected known inhibitors of NF-α (TNFI)

Table S2. Their physicochemical properties of Selected TNFIs

Figure S1. (A). The docked conformation of SPD obtained by FRED, AutoDock Vina, MOE and MVD, respectively. The X-ray and Re-docked orientations of SPD are shown in red and magenta stick models, respectively (B) The re-docked orientation of JNJ525 obtained by MOE, FRED, AutoDock Vina, and MVD. The experimental and docked conformations are presented in yellow and green stick model, respectively.

Figure S2. Percent Enrichment Factor (%EF) calculated for four docking programs on 2AZ5

Figure S3. Receiver Operating Curve (ROC) for 2AZ5 obtained from FRED, MOE AutoDock Vina, and MVD. AUC for FRED, AutoDock Vina, MOE and MVD. The AUC value of 0.95, 0.86, 0.92 and 0.87 was observed for FRED, MOE, Autodock Vina, and MVD, respectively.

Figure S4. Superimposed view of Compounds TNFI2, TNFI5, TNFI6, TNFI22, TNFI24 and TNFI39 on LBPM1 and superimposed view of twenty Compounds (TNFI1-11, TNFI15, TNFI20-25, TNFI34 and TNFI41) on LBPM2

Table S3. Pharmacokinetic and Physiological Properties of 17 selected hits.
Interactions Analysis of Compounds 1-3, 6, 8, and 10-17

Figure S5. The docked view of compounds 1-3, 6, 8, 10-17. The interacting residues of Chain A and B are shown in coral and yellow colour, respectively. The ligands are depicted in magenta stick model. Hydrogen bonds are displayed in green lines. H-bond distances are written in red colour. Compounds numbers are written in parenthesis.

Table S1. The chemical structures and biological activities of selected known inhibitors of TNF-α (TNFI)

TNF Inhibitors (TNFI)	Structures	IC$_{50}$ (μM)	Reference
**1**	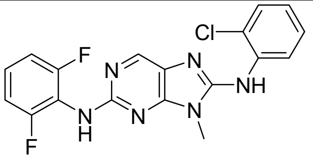	1.5	1
**2**	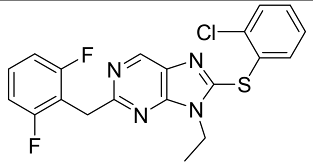	0.014	1
**3**	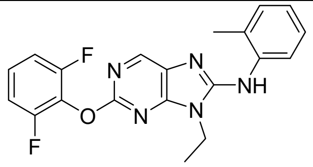	0.048	1
**4**	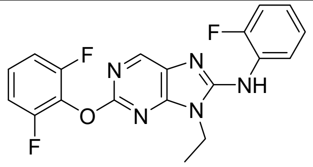	0.035	1
**5**[†]	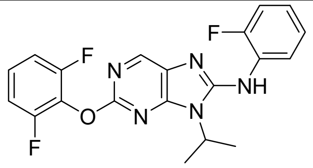	0.004	1
**6**	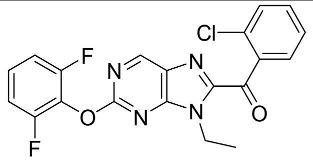	0.004	1
**7**	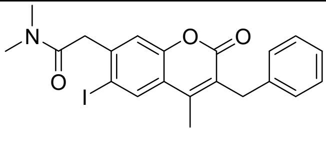	0.06	2
**8**	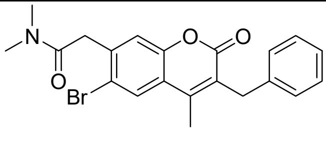	0.06	2

**9**[†]	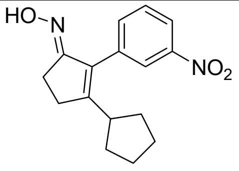	0.24	3
**10**	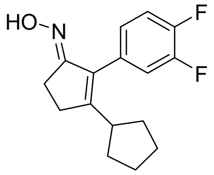	0.26	3
**11**	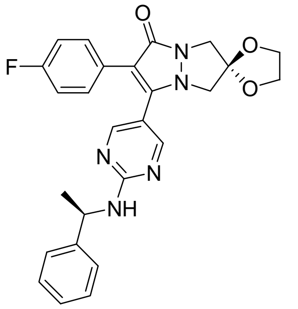	2	4
**12**	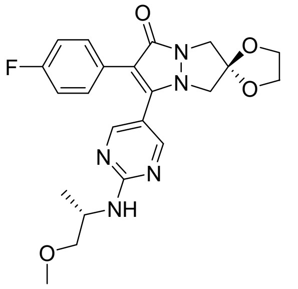	36	4
**13**	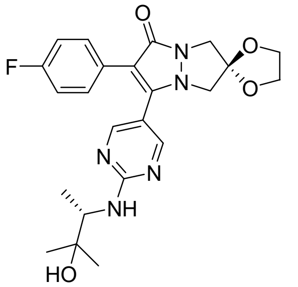	9	4
**14**	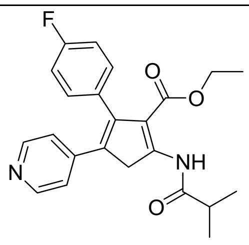	36	5

**15**	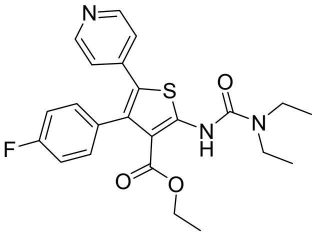	2.6	5
**16**	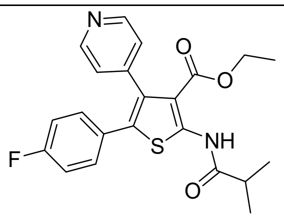	5.4	5
**17**	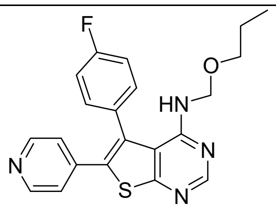	10	5
**18**	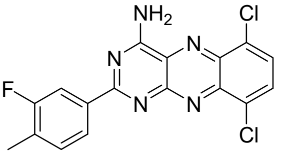	5.8	6
**19**	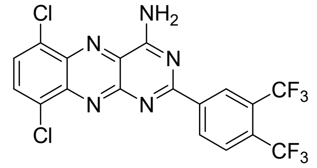	7.3	6
**20**	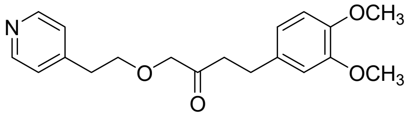	2	7
**21**	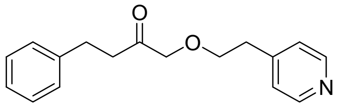	3	7
**22**	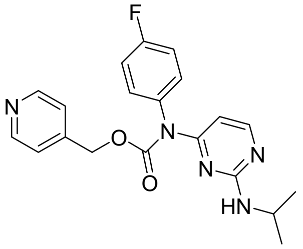	0.004	8

**23**	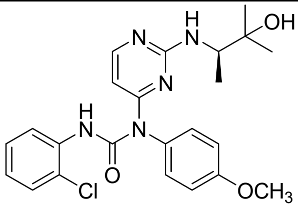	0.092	8
**24**†	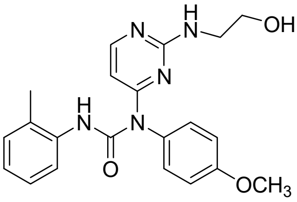	0.009	8
**25**	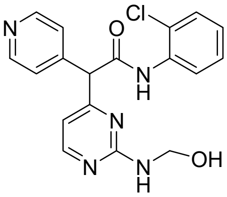	0.071	8
**26**	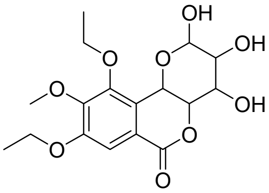	31	9
**27**	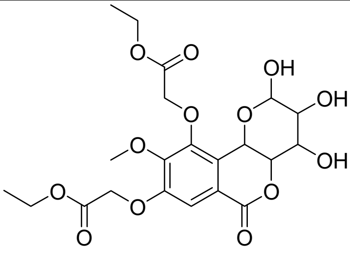	60	9
**28**	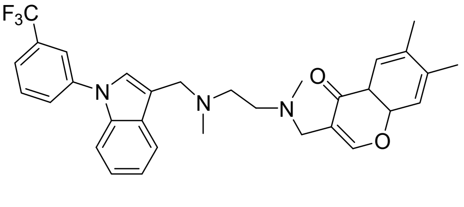	22	10

**29**	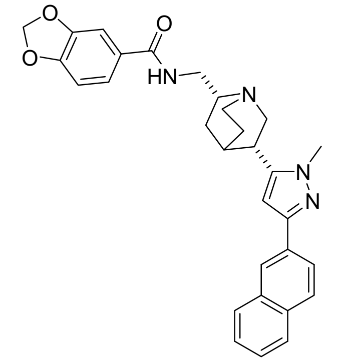	5	10
**30**	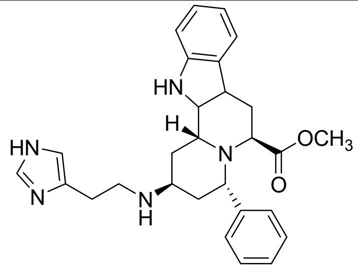	30	10
**31**	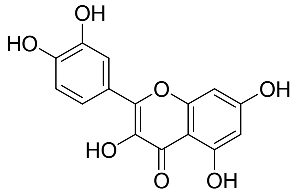	50	10
**32**	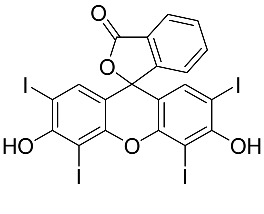	50	10
**33**	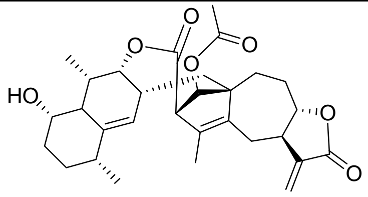	10	10
**34**	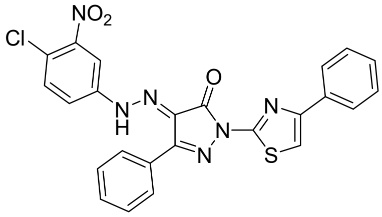	10	10

**35**	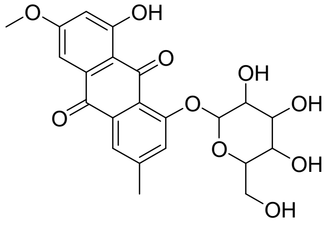	20	10
**36**	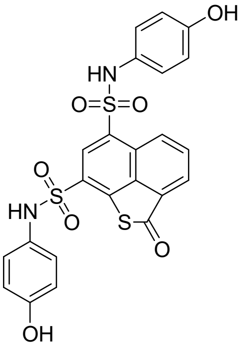	14	11
**37**	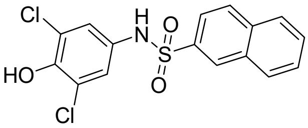	>100	11
**38**	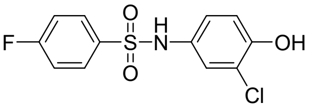	>100	11
**39**[†]	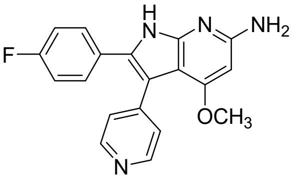	0.0063	12
**40**	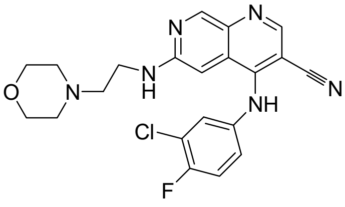	6.8	13
**41**[†]	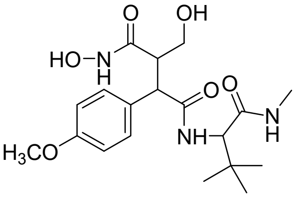	0.048	14

**42**	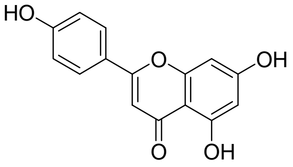	27.4	15

ǂ ***Compounds used in 2D-Similarity searching***

Compounds TNFI2, TNFI5, TNFI6, TNFI22, TNFI24 and TNFI39 were used in the generation of LBPM1

Compounds TNFI1-11, TNFI15, TNFI20-25, TNFI34 and TNFI41 were used in the generation of LBPM2

Table S2. Their physicochemical properties of Selected TNFIs

Compounds	MW	TPSA	logP(o/w)	HBA	HBD	RB
**1**	386.7930	67.6600	4.2941	3	2	4
**2**	416.8830	43.6000	5.8321	3	0	5
**3**	381.3860	64.8600	4.5691	3	1	5
**4**	385.3490	64.8600	4.4241	3	1	5
**5**	399.3760	64.8600	4.8861	3	1	5
**6**	414.7990	69.9000	4.6631	4	0	5
**7**	461.2990	46.6100	4.9890	2	0	5
**8**	414.2990	46.6100	4.5970	2	0	5
**9**	286.3310	78.4100	3.6460	2	1	3
**10**	277.3140	32.5900	4.0150	2	1	2
**11**	473.5080	79.8200	3.1020	5	1	5
**12**	441.4630	89.0500	1.2320	6	1	6
**13**	455.4900	100.0500	1.4890	6	2	5
**14**	394.4460	68.2900	4.0850	3	1	8
**15**	441.5270	71.5300	4.5790	3	1	10
**16**	412.4850	68.2900	4.9010	3	1	8
**17**	394.4740	59.9300	4.5760	4	1	7
**18**	374.2060	77.5800	4.5476	4	1	1
**19**	478.1830	77.5800	5.9661	4	1	3
**20**	329.3960	57.6500	2.0697	5	0	10
**21**	269.3440	39.1900	2.3710	3	0	8
**22**	381.4110	80.2400	2.8890	4	1	8
**23**	455.9460	99.6100	3.5720	5	3	9
**24**	393.4470	99.6100	1.9470	5	3	9
**25**	369.8120	100.0300	0.9650	5	3	7
**26**	370.3540	123.9100	-0.0845	8	3	5
**27**	486.4260	176.5100	-1.0430	10	3	11
**28**	549.6370	37.7100	5.5668	4	0	9
**29**	494.5950	68.6200	4.2630	5	1	6
**30**	471.6050	82.2800	3.1580	5	4	7
**31**	302.2380	127.4500	2.0320	6	5	1
**32**	835.8950	75.9900	8.8740	3	2	0
**33**	522.6380	99.130	1.8390	4	1	2
**34**	502.9420	115.7700	8.1920	4	1	6
**35**	446.4080	162.9800	0.4820	10	5	4
**36**	528.5860	149.8700	4.3020	7	4	6
**37**	368.2400	66.4000	4.8600	3	2	3
**38**	301.7250	66.4000	3.1270	3	2	3
**39**	334.3540	76.8200	3.7770	4	3	3
**40**	426.8830	86.1000	2.4195	5	2	7
**41**	395.4560	136.9900	0.1430	6	5	12
**42**	270.2400	86.9900	2.5340	4	3	1

MW = Molecular weight, TPSA = Topological polar surface area, logP(o/w) = logP(octanol/water), HBD = Hydrogen Bond Donors, HBA = Hydrogen Bond Acceptors, RB = Number of Rotatable Bonds

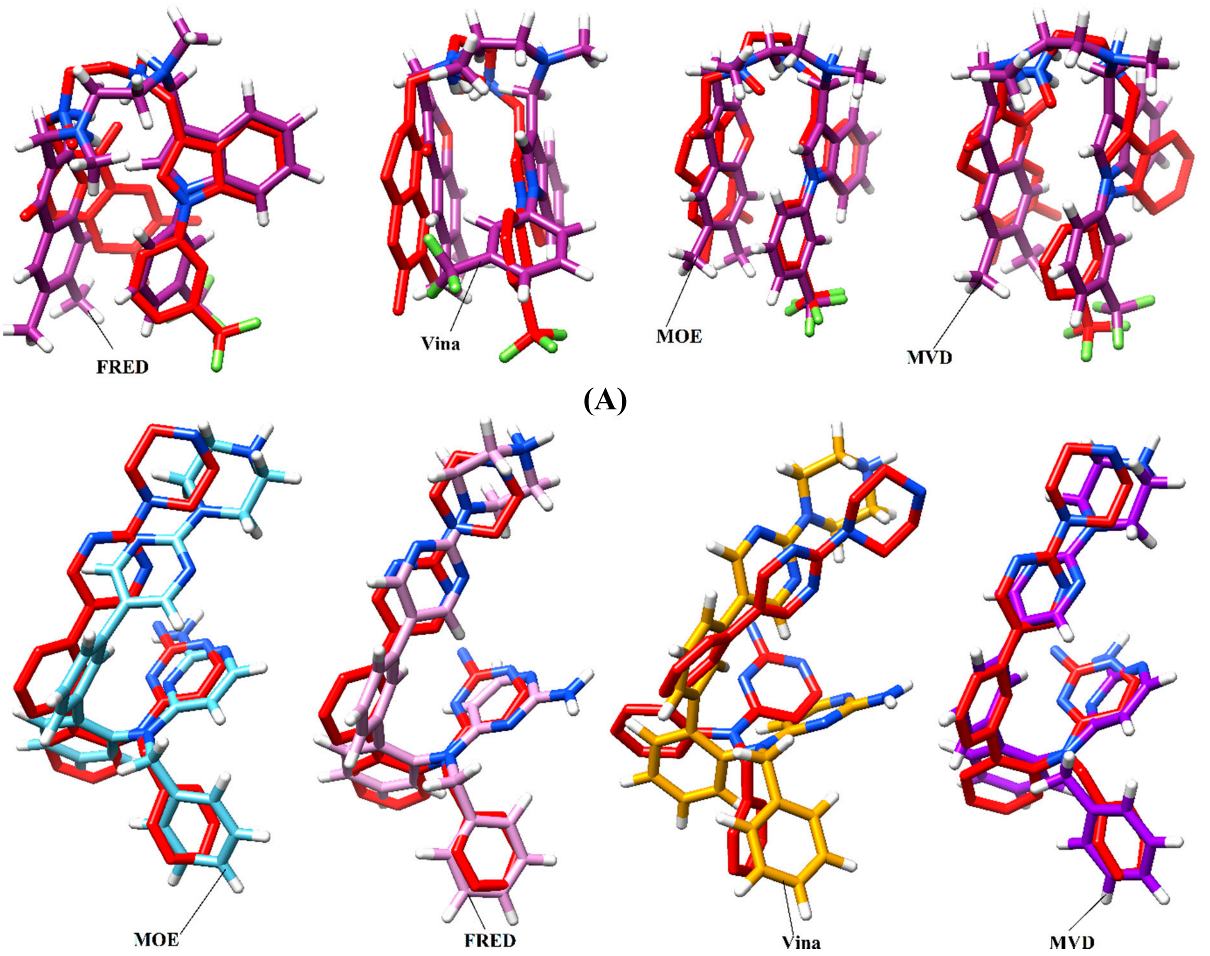

(B)

Figure S1. (A). The docked conformation of **SPD** obtained by FRED, AutoDock Vina, MOE and MVD, respectively. The X-ray and Re-docked orientations of **SPD** are shown in red and magenta stick models, respectivley (B) The re-docked orientation of **JNJ525** obtained by MOE, FRED, AutoDock Vina, and MVD. The experimental and docked conformations are presented in yellow and green stick model, respectively.

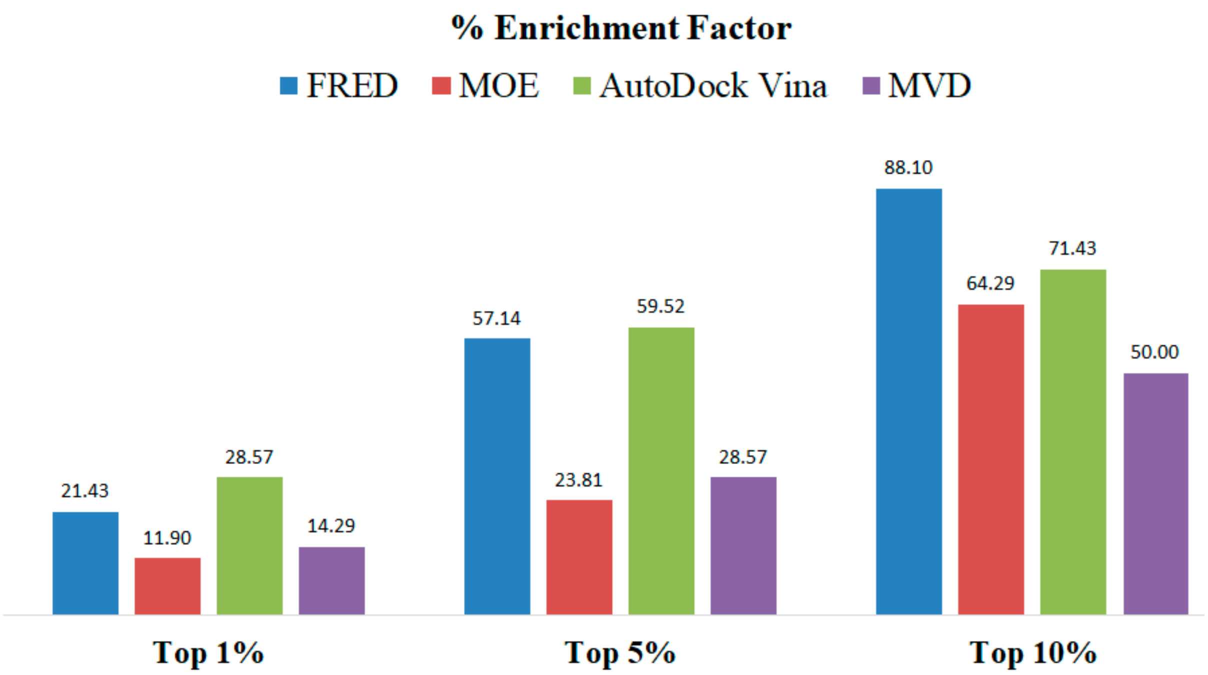

Figure S2. Percent Enrichment Factor (%EF) calculated for four docking programs on 2AZ5

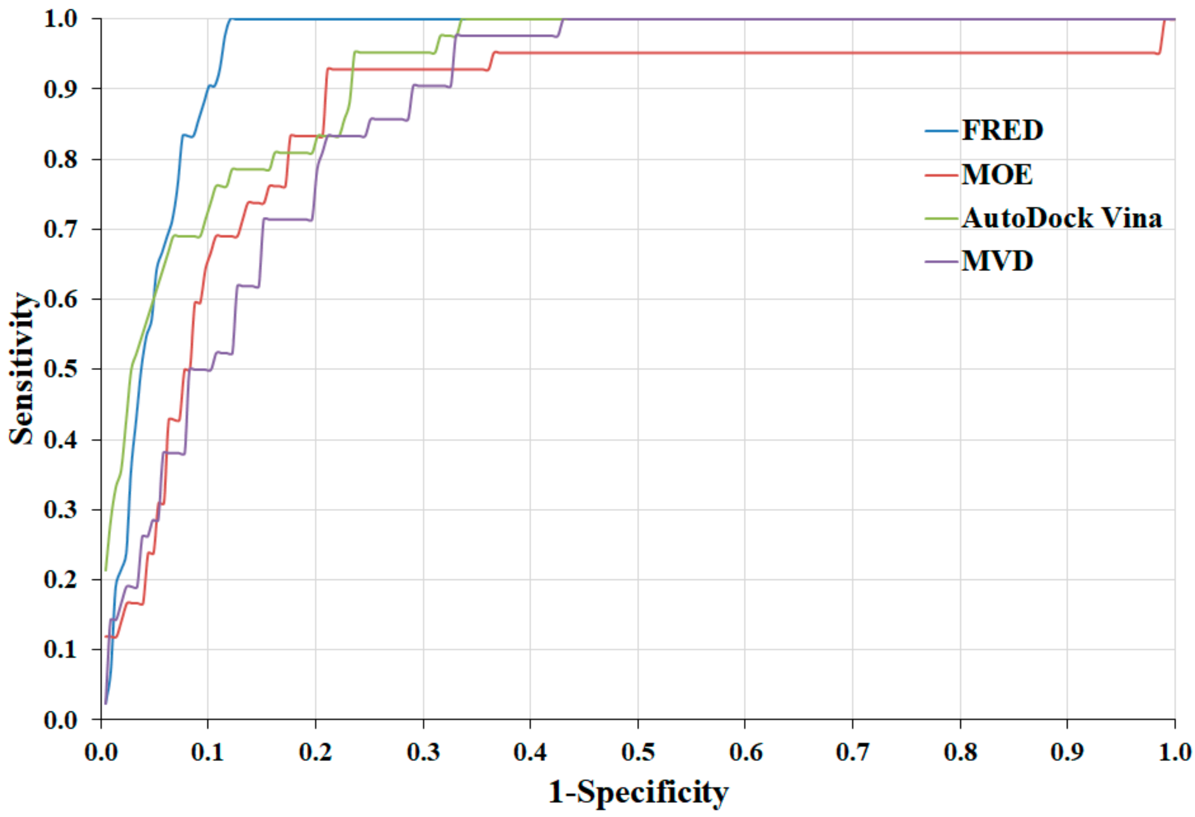

Figure S3. Receiver Operating Curve (ROC) for 2AZ5 obtained from FRED, MOE AutoDock Vina, and MVD. AUC for FRED, AutoDock Vina, MOE and MVD. The AUC value of 0.95, 0.86, 0.92 and 0.87 was observed for FRED, MOE, Autodock Vina, and MVD, respectively.

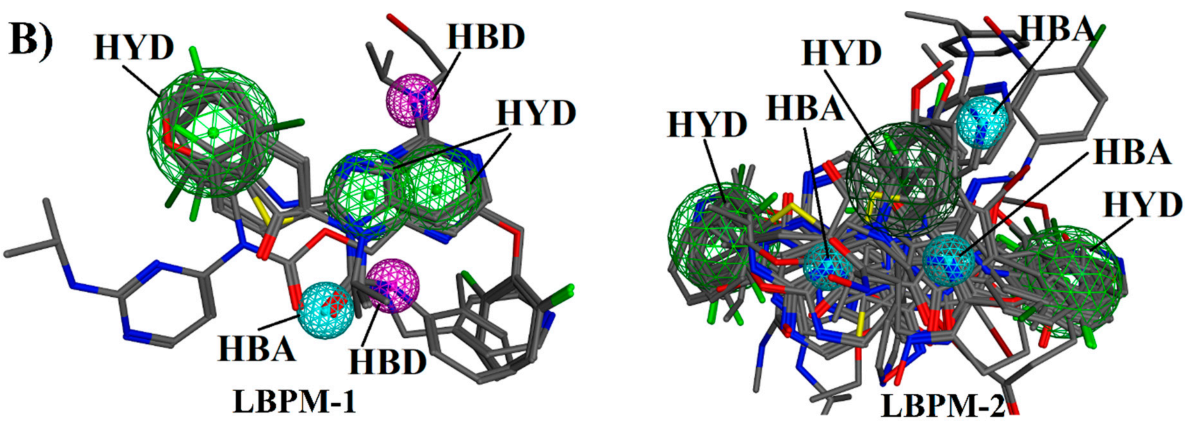

Figure S4. Superimposed view of Compounds TNFI2, TNFI5, TNFI6, TNFI22, TNFI24 and TNFI39 on LBPM1 and superimposed view of twenty Compounds (TNFI1-11, TNFI15, TNFI20-25, TNFI34 and TNFI41) on LBPM2

Table S3. Pharmacokinetic and Physiological Properties of 17 selected hits.

Comp #	Pharmacokinetics			AMES toxicity	Acute Oral Toxicity	Carcinogenicity	Rat Acute Toxicity [LD_{50}, mol/kg]	Physicochemical Properties	Drug Likeness	SA
	HIA	BBB	CYP inhibition/substrate							
**1**	High	Yes	P-gpS: Yes, NS: CYP2C9, 2D6, S: CYP3A4, I: CYP1A2, 2C9, 2C19, 3A4, NI: CYP2D6, Log K_p: -6.22 cm/s	None	III	None	2.36	F:C21H16ClF2NO2, MW:387.81g/mol HA:27, RB:2, HBA:4, HBD:1, TPSA:46.17Å², Log*P*o/w: 2.66, Log *S*: -4.61 (MS)	Yes	4.25
**2**	High	Yes	P-gpS: Yes, NS: CYP2C9, 2D6, S: CYP3A4, NI: CYP1A2, 2D6, I: CYP2C9, 3A4, Log K_p: -6.37 cm/s	None	III	None	2.75	F:C23H20F3NO3, MW: 415.41 g/mol, HA: 30, RB: 4, HBA: 6, HBD: 1, TPSA: 55.40Å², Log*P*o/w: 2.74, LogS: -4.63(MS)	Yes	4.35
**3**	High	Yes	P-gpS: Yes, NS: CYP2C9, 2D6 S: CYP3A4, I: CYP2C9, 2C19,3A4, NI: CYP1A2,2D6, Log K_p: -6.82 cm/s	None	III	None	2.61	F: C23H22FNO4, MW:395.42 g/mol, HA: 29, RB: 4, HBA: 5, HBD: 1, TPSA:64.63Å², Log*P*o/w: 2.66, LogS: -4.01(MS)	Yes	4.29
**4**	High	Yes	P-gpS: No, NS: CYP2C9,2D6 S: CYP3A4, I: CYP2C9, 2C19,3A4, I:CYP1A2,2D6, Log K_p: -7.04 cm/s	None	III	None	2.22	F:C23H19ClFN3O3, MW: 439.87 g/mol, HA: 31, RB:1, HBA: 5, HBD: 1, TPSA: 69.72Å², Log*P*o/w: 3.03, LogS: -4.51 (MS)	Yes	4.58
**5**	High	Yes	P-gpS: No, NS: CYP2C9,2D6 S: CYP3A4 I: CYP2C19, 3A4	None	III	None	2.33	F: C25H23ClN2O4,MW:450.91g/mol HA: 32, RB: 2, HBA: 5, HBD: 0, TPSA: 66.92Å², Log*P*o/w: 3.61, LogS: -4.76 (MS)	Yes	4.67

			NI: CYP1A2,2C9, 2D6, Log K_p: -6.82 cm/s							
**6**	High	Yes	P-gpS: Yes, NS: CYP2C9,2D6, S: CYP3A4, I: CYP2C9, 2C19, 3A4, NI: CYP1A2,2D6, Log K_p: -5.87 cm/s	None	III	None	2.78	F:C25H24N2O3, MW: 400.47 g/mol, HA:30, RB:2, HBA:4, HBD:2, TPSA: 71.45 Å², Log*P*o/w: 3.26, LogS: -5.14 (MS)	Yes	4.16
**7**	High	Yes	P-gpS: Yes, NS: CYP2C9, 2D6 S: CYP3A4, I: CYP1A2, 2C9, 2C19, 3A4, NI: CYP2D6 Log K_p:-6.51 cm/s	None	III	None	2.53	F:C26H21FN4O3, MW:456.47 g/mol, HA: 34, RB:4, HBA: 6, HBD: 1, TPSA:73.66Å², Log*P*o/w: 3.04, LogS: -5.19 (MS)	Yes	4.99
**8**	High	No	P-gpS: Yes, NS: CYP2C9, 2D6, S:CYP3A4, I:CYP1A2,2C9, 2C19, 3A4, NI: CYP2D6, Log K_p:-7.19 cm/s	None	III	None	2.53	F:C22H21N5O3, MW: 403.43g/mol HA: 30, RB: 4, HBA: 6, HBD: 1 TPSA: 91.16 Å², Log*P*o/w: 2.70, LogS: -3.89 (Soluble)	Yes	4.61
**9**	High	No	P-gpS: Yes, NS: CYP2C9, 2D6 S: CYP3A4, I: CYP2C9, 2D6, 2C19, 3A4, NI: CYP1A2, Log K_p:- 7.54 cm/s	None	III	None	2.32	F: C25H22ClN3O5, MW: 479.91 g/mol, HA: 34, RB:3, HBA: 6, HBD: 1, TPSA: 96.02 Å², Log*P*o/w: 3.22, LogS: -4.38 (MS)	Yes	4.81
**10**	High	Yes	P-gpS: No, NS: CYP2C9, 2D6 S: CYP3A4, I:CYP1A2,2C9, 2C19, 3A4, NI: CYP2D6, Log K_p:- 6.44cm/s	Yes	III	None		F:C18H21N4O, MW: 309.39 g/mol HA: 23, RB:6, HBA: 3, HBD: 1 TPSA: 67.58 Å², Log*P*o/w: 2.83, LogS: -3.46 (Soluble)	Yes	3.28

**11**	High	Yes	P-gpS: No, NS: CYP2C9, 2D6, 3A4, I: CYP1A2, 2C19, NI: CYP2C9, 2D6, 3A4, Log K_p:-5.37 cm/s	None	III	None	2.53	F:C16H18F3NO, MW: 297.32 g/mol HA: 21, RB: 6, HBA: 4, HBD: 1, TPSA: 29.10 Å², Log*P*o/w: 2.97, LogS: -3.93 (MS)	Yes	2.89
**12**	High	Yes	P-gpS: Yes, NS: CYP2C9, 3A4 S: CYP2D6, I: CYP1A2, 2C9, 2C19, 3A4, NI: CYP2D6, Log K_p:-6.46 cm/s	None	III	None	2.76	F:C19H17N3OS, MW: 335.42 g/mol HA: 24, RB: 3, HBA: 3, HBD: 0 TPSA: 71.39 Å², Log*P*o/w: 2.63, LogS: -3.89 (Soluble)	Yes	2.51
**13**	High	No	P-gpS: No, NS: CYP450 2C9, 2D6, 3A4 NI: CYP450 1A2, 2C9,2D6,2C19,3A4 Log K_p:-6.52 cm/s	None	III	None	2.19	F:C17H13FN2O3, MW: 312.30 g/mol, HA: 23, RB: 3, HBA: 4, HBD: 2, TPSA: 75.27 Å², Log*P*o/w:1.47, LogS: -3.46 (Soluble)	Yes	2.59
**14**	High	No	P-gpS: No, NS: CYP2C9, 2D6 S: CYP3A4, I: CYP1A2, 2C9, 2C19, 3A4, NI: CYP2D6 Log K_p:-6.01 cm/s	None	III	None	2.54	F:C22H16ClN3O4S, MW: 453.90 g/mol, HA: 31, RB: 5, HBA: 5, HBD: 1, TPSA: 113.90 Å², Log*P*o/w: 3.05, LogS: -5.42 (MS)	Yes	3.39
**15**	High	Yes	P-gpS: No, NS: CYP2C9, 2D6, 3A4, I: CYP1A2, 2C9, 2D6, 2C19, 3A4, Log K_p:-5.08 cm/s	Yes	III	None	2.60	F:C21H15N3O, MW: 325.36 g/mol HA: 25, RB: 3, HBA: 3, HBD: 1, TPSA: 61.70 Å², Log*P*o/w: 2.74, LogS: -5.07 (MS)	Yes	2.54
**16**	High	Yes	P-gpS: Yes, NS: CYP2C9, 2D6 S: CYP3A4, I: CYP2D6, NI: CYP450 1A2, 2C9, 2C19, 3A4	None	III	None	2.65	F:C22H29N2O2S, MW: 385.54 g/mol HA: 27, RB:5, HBA: 2, HBD: 2, TPSA: 62.44 Å², Log*P*o/w: 3.77, LogS: -5.10 (MS)	Yes	4.28

			Log K_p:-5.42 cm/s							
**17**	High	Yes	P-gpS: Yes, NS: CYP2C9, 2D6 S: CYP3A4, I: CYP2C19, 3A4, 2C9, NI: CYP1A2, 2D6 Log K_p:-6.61 cm/s	None	III	None	2.70	F:C24H25NO4, MW: 391.46 g/mol HA: 29, RB:4, HBA: 4, HBD: 1, TPSA: 64.63 Å², Log*P*o/w: 3.05, LogS: -4.15(MS)	Yes	4.45

HIA: Human Intestinal Absorption, BBB: Blood Brain Barrier, P-gpS: P-Glycoprotein substrate, I = Inhibitor, NI = Non-inhibitor, S = Substrate, NS = non-substrate, CIP: CYP Inhibitory Promiscuity, Log K_p =skin permeation, F= Formula, MW= Molecular Weight, HA = Number of Heavy Atoms, RB = Number of Rotatable bonds, HBA = Number of H-bond acceptors, HBD = Number of H-bond donors, TPSA = Topological Polar Surface Area, Log*P*o/w= Lipophilicity, Log *S*= Water Solubility, MS= Moderately soluble, Log K_p = Skin Permeation, III: Category III includes compounds with LD_{50} values >500mg/kg but <5000mg/kg, SA = Synthetic Accessibility

Interactions Analysis of Compounds 1-3, 6, 8, and 10-17

The binding mode of compound 1 revealed that the quinolone nitrogen and the quinolone carbonyl of 1 mediates H-bonding with Gly121A (1.9Å) and Tyr151B (2.5Å), respectively. Moreover, several residues including Tyr59A, Gln61A, Tyr119A, Tyr151A, Tyr59B, and Tyr119B provide hydrophobic (HYD) interactions thus support the fitting of the compound between chains A and B. These interactions suggest that the compound hinder the binding of the TNF-α receptor at the TNF-α/TNFR binding interface.

The hydro-quinolinedione of compound 2 mediates bi-dentate interactions with Tyr51B (2.70Å) and Gly121A (2.13Å). A strong π-π interaction is observed between tri-fluoro substituted phenyl ring and Tyr119B. Moreover, hydroquinolinedione moiety is hydrophobically stabilized by Tyr59B. Hence both H-bond and hydrophobic interactions collectively stabilize the compound in the binding region.

The hydroquinolinedione moiety of compound 3 interacts with both chains A and B. The quinoline –NH3 donates H-bond to the carbonyl of Gly121A (1.87Å) while quinoline -carbonyl accepts H-bond from –OH of Tyr151B (2.38Å). Additionally, dimethoxy substituted phenyl ring mediate CH3-π interactions with the phenyl of Tyr59A.

Compound 6 neatly fits in the ligand binding site and forms H-bond with –OH of Tyr151A (3.0Å). Additionally, Tyr59A provides strong π- π interactions to the pyridine moiety of the compound. Similarly, Leu57B stabilizes the compound via HYD interactions. Moreover, chain B of TNF-α provides several HYD interactions to the methoxy phenol of the compound. Compound 6 interacted with –OH of Tyr151B. The dimethoxy phenyl mediates H-bond with –OH of Tyr151B (3.3Å). Moreover, fluorophenyl mediates strong π-π interactions with Tyr119A.

The compound 8 forms multiple H-bonds with the receptor binding site of TNF-α. The phenyl-substituted acetate interacts with the –OH of Tyr151A (2.5Å) while pyrrolidinone NH3 donates H-bond to peptide carbonyl (1.97Å). Similarly, the carbonyl of pyrrolidinone ring accepts H-bond from the –OH of Tyr151B (2.73Å).

The peptide-NH3 of compound 10 donates H-bond to the carbonyl of Gly121A (1.82Å). This compound mainly interacts with Chain A of TNF-α. Moreover, the hydrocarbon ring interacts with Leu57A, Leu57B and Tyr59B.

Compound 11 is stabilized by two H-bonds with both chains. The peptide carbonyl accepts H-bond from the –OH of Tyr151B (3.30Å), while the quinoline substituted pyridine ring accepts H-bond from –OH of Tyr151A (2.60Å). The H-bond distances shows that the orientation of compound is more towards the chain A, while weak H-bonds can exist between compound and chain B.

Compound 12 interacts with the chain B via two H-bonds. The pyrimidine trione-NH3 formed H-bonds with carbonyl of Leu120B and –OH of Tyr151B at a distance of 2.40Å and 2.37Å, respectively. However, phenyl and the fluorophenyl ring is tilted towards Tyr59B and chain A, respectively.

Compound 13 mediates H-bonding with the chain B while interacts hydrophobically with chain A. The thioxo-imidazolidinone –NH3 of 14 forms H-bond with carbonyl of Ser60B (2.08Å). While –OH of Tyr151B donates H-bond to the carbonyl of thioxo-imidazolidinone ring (3.0Å). The quinoline substituted chlorophenyl ring forms HYD interaction with Leu57B.

The compound 15 interacts with chain A via H-bonds. The benzo-imidazole –NH3 is H-bonded to the carbonyl of Gly121 (1.80Å). The diphenyl substituted methoxy group accepts H-bond from –OH of Tyr151A (3.05Å). The benzo-imidazole ring fits towards chain B, while diphenyl ring is tilted towards chain A.

The piperidinol ring and phenothiazine moiety of compound 16 is oriented towards the chain A and B, respectively. The piperidinol is H-bonded to–OH of Tyr151A (2.76Å). The phenothiazine moiety mainly faces Leu57B, Tyr59B, Gly121B, and Gly122B. Hence both HYD and H-bonding stabilize the compound at this interface.

The -NH3 and carbonyl of quinoline-dione moiety of compound 17 interact with the carbonyl of Gly121A and–OH of Tyr151B with the distance of 2.21Å and 2.33Å, respectively. The quinoline substituted phenyl ring is oriented towards Tyr119A and Tyr119B. The binding modes of the selected hits are depicted in Figure S4. The H-bond distances are written in figure.

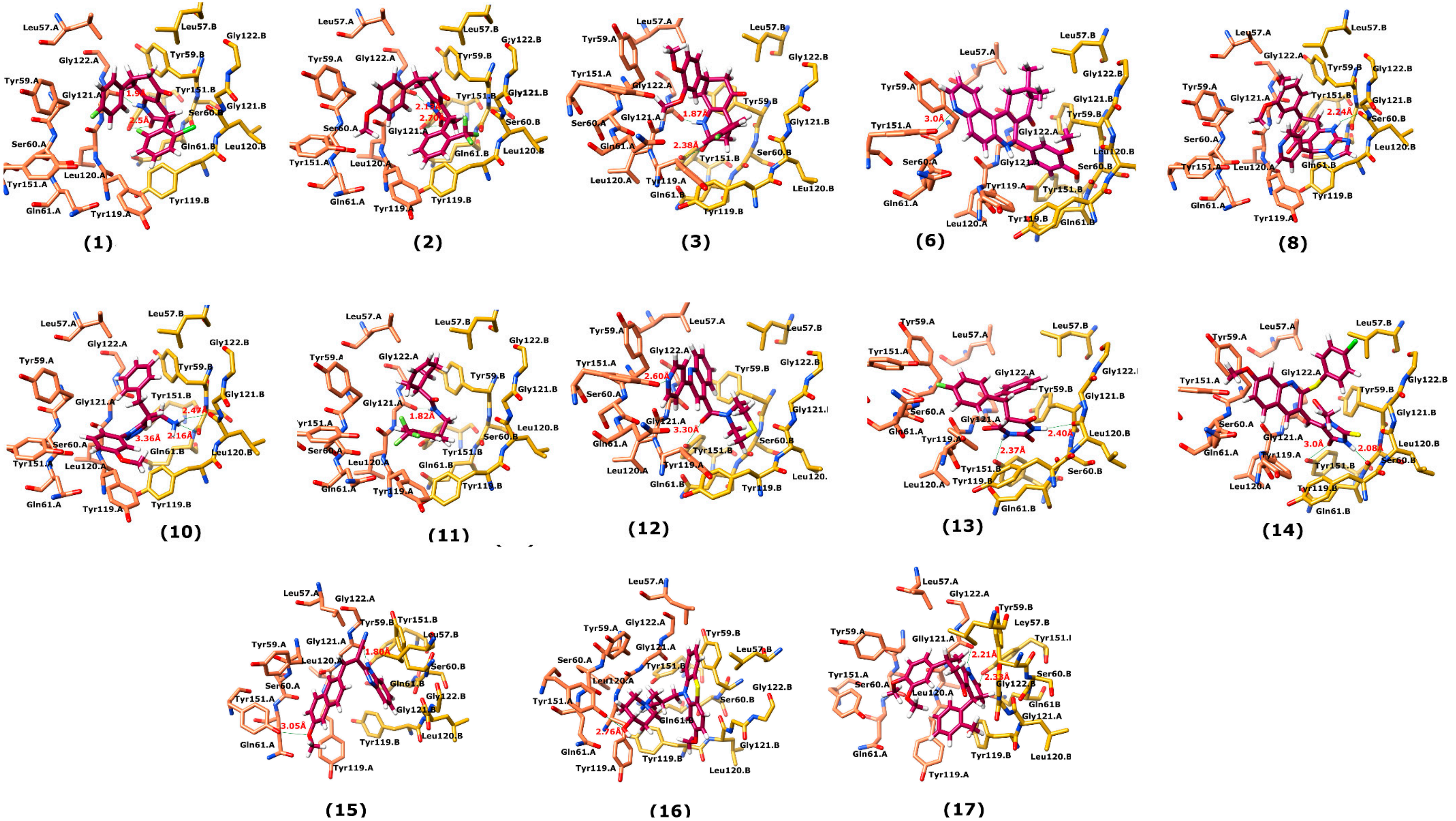

Figure S5. The docked view of compounds 1-3, 6, 8, 10-17. The interacting residues of Chain A and B are shown in coral and yellow colour, respectively. The ligands are depicted in magenta stick model. Hydrogen bonds are displayed ingreen lines. H-bond distances are written in red colour. Compounds numbers are written in parenthesis.

References

1. Sabat, M., et al. The development of novel C-2, C-8, and N-9 trisubstituted purines as inhibitors of TNF-a production. *ChemInform*, **2006**, *37*, 4360–4365.
2. Cheng, J. F., et al. Discovery and structure-activity relationship of coumarin derivatives as TNF-a inhibitors. *ChemInform*, **2004**, *35*, 2411–2415.
3. Kim, Y., et al. Synthesis and structure-activity relationship of cyclopentenone oximes as novel inhibitors of the production of tumor necrosis factor-α. *Bioorg. Med. Chem. Lett.* **2014**, *24*, 2807–2810.
4. Laufersweiler, M., et al. The development of novel inhibitors of tumor necrosis factor-a (TNF-a) production based on substituted [5,5]-bicyclic pyrazolones. *ChemInform*, **2004**, *35*, 4267–4272.
5. Fujita, M., Hirayama, T., and Ikeda, N. Design, synthesis, and bioactivities of novel diarylthiophenes: Inhibitors of tumor necrosis factor-a (TNF-a) production. *Bioorg. Med. Chem.* **2002**, *10*, 3113–3122.
6. Guirado, A., et al. First synthesis and biological evaluation of 4-amino-2-aryl-6,9-dichlorobenzo[g]pteridines as inhibitors of TNF-a and IL-6. *Eur. J. Med. Chem.* **2013**, *66*, 269–275.
7. Dhuru, S., et al. Novel diarylheptanoids as inhibitors of TNF-a production. *Bioorg. Med. Chem. Lett.* **2011**, *21*, 3784–3787.
8. Mouchlis, V., et al. Molecular modeling on pyrimidine-urea inhibitors of TNF-a production: An integrated approach using a combination of molecular docking, classification techniques, and 3D-QSAR CoMSIA. *J. Chem. Inf. Model.* **2012**, *52*, 711–723.
9. Shah, M. R., et al. Synthesis of new bergenin derivatives as potent inhibitors of inflammatory mediators NO and TNF-a. Bioorg. *Med. Chem. Lett.* **2012**, *22*, 2744–2747.
10. Chen, S., et al. Discovery of Novel Ligands for TNF-α and TNF Receptor-1 through Structure-Based Virtual Screening and Biological Assay. *J. Chem. Inf. Model.* **2017**, *57*, 1101-1111.
11. Shen, Qi., et al. Discovery of highly potent TNFα inhibitors using virtual screening. *Eur. J. Med. Chem,* **2014**, *85*, 119-126
12. Henry, J. R., et al. Potent inhibitors of the Map Kinase p83. *Bioorg. Med. Chem. Lett.* **1998**, 3335-3340.
13. Gavrin, L. L., et al. Inhibition of Tpl2 kinase and TNF-alpha production with 1,7-naphthyridine-3-carbonitriles: synthesis and structure-activity relationships. *Bioorg. Med. Chem. Lett.* **2005**, *15*, 5288-92.
14. Kottirsch, G., et al. Beta-aryl-succinic acid hydroxamates as dual inhibitors of matrix metalloproteinases and tumor necrosis factor alpha converting enzyme. *J Med Chem.* **2002,** *45,*2289-93.
15. Seo, H-S., et al. Apigenin Inhibits Tumor Necrosis Factor-α-Induced Production and Gene Expression of Mucin through Regulating Nuclear Factor-Kappa B Signaling Pathway in Airway Epithelial Cells. *Biomol. Ther.* **2014**, *22*, 525-31.
